# Physician Presence in an Ambulance Car Is Associated with Increased Survival in Out-of-Hospital Cardiac Arrest: A Prospective Cohort Analysis

**DOI:** 10.1371/journal.pone.0084424

**Published:** 2014-01-08

**Authors:** Akihito Hagihara, Manabu Hasegawa, Takeru Abe, Takashi Nagata, Yoshihiro Nabeshima

**Affiliations:** 1 Department of Health Services Management and Policy, Kyushu University Graduate School of Medicine, Fukuoka, Japan; 2 Ambulance Service Planning Division, Fire and Disaster Management Agency, Ministry of Internal Affairs and Communications, Tokyo, Japan; 3 Faculty of Human Sciences, Waseda University, Tokorozawa, Japan; 4 Department of Emergency and Critical Care Center, Kyushu University Hospital, Fukuoka, Japan; San Raffaele Scientific Institute, Italy

## Abstract

The presence of a physician seems to be beneficial for pre-hospital cardiopulmonary resuscitation (CPR) of patients with out-of-hospital cardiac arrest. However, the effectiveness of a physician's presence during CPR before hospital arrival has not been established. We conducted a prospective, non-randomized, observational study using national data from out-of-hospital cardiac arrests between 2005 and 2010 in Japan. We performed a propensity analysis and examined the association between a physician's presence during an ambulance car ride and short- and long-term survival from out-of-hospital cardiac arrest. Specifically, a full non-parsimonious logistic regression model was fitted with the physician presence in the ambulance as the dependent variable; the independent variables included all study variables except for endpoint variables plus dummy variables for the 47 prefectures in Japan (*i.e.*, 46 variables). In total, 619,928 out-of-hospital cardiac arrest cases that met the inclusion criteria were analyzed. Among propensity-matched patients, a positive association was observed between a physician's presence during an ambulance car ride and return of spontaneous circulation (ROSC) before hospital arrival, 1-month survival, and 1-month survival with minimal neurological or physical impairment (ROSC: OR = 1.84, 95% CI 1.63–2.07, *p* = 0.00 in adjusted for propensity and all covariates); 1-month survival: OR = 1.29, 95% CI 1.04–1.61, *p* = 0.02 in adjusted for propensity and all covariates); cerebral performance category (1 or 2): OR = 1.54, 95% CI 1.03–2.29, *p* = 0.04 in adjusted for propensity and all covariates); and overall performance category (1 or 2): OR = 1.50, 95% CI 1.01–2.24, *p* = 0.05 in adjusted for propensity and all covariates). A prospective observational study using national data from out-of-hospital cardiac arrests shows that a physician's presence during an ambulance car ride was independently associated with increased short- and long-term survival.

## Introduction

The presence of a physician before hospital arrival is believed to lead to effective cardiopulmonary resuscitation (CPR) for patients with out-of-hospital cardiac arrest (OHCA) [1]–[3]. Several studies have described ambulance crews staffed with a physician including cardiac ambulance crews [4], helicopter ambulance teams [5] and physician-manned ambulance (PMA) teams comprised of two paramedics and an anesthesiologist [6]. However, findings concerning whether the presence of a physician before hospital arrival leads to improved patient outcome during CPR are mixed.

A randomized, controlled trial in the 1980s showed that the mortality rate in a group of patients for whom a physician was present was 35% lower than predicted, while the mortality in the non-physician group was as predicted [5]. It has been pointed out, however, that the study design might have carried inherent bias [7]. A study performed in Nottingham, England reported a trend towards improved survival in patients treated by PMA teams. However, the study might have been influenced by selection bias since the subjects were not randomized [6]. In addition, it is unclear how the results of this 1970s study might apply to the current emergency medical service (EMS) system. A study performed in Taiwan reported improved survival in patients treated by non-PMA teams. However, the overall survival rate was only 1.4%, and the validity of the study has been questioned [8]. According to a systematic review of non-randomized studies between 1990 and 2008, four of five studies in patients with out-of-hospital cardiac arrest (OHCA) revealed a significantly higher survival rate in the PMA group than in the non-PMA group [7]. However, these studies were limited by several methodological issues, such as the use of samples from a single center and type II errors due to the inclusion of fewer than 100 patients [9]–[13].

In these previous studies, the findings are inconsistent and the effectiveness of a physician's presence during pre-hospital CPR has yet to be established. To verify the effectiveness of the presence of a physician during CPR of patients with OHCA before hospital arrival, the influences of other factors, such as patients, bystanders, and CPR, need to be controlled. Although a randomized, controlled study should be performed, such a study is challenging because of ethical reasons. Therefore, using a propensity analysis, we analyzed OHCA cases using national data in Japan from 2005 to 2010 and determined whether the presence of a physician in the ambulance was associated with immediate and 1-month survival in patients with OHCA.

## Methods

### Study design

This was a prospective observational study using national registry data. The subjects were patients who were 18 years of age or older, had OHCA before the arrival of EMS personnel, were treated by EMS personnel and were then transported to medical institutions in Japan between 1 January 2005 and 31 December 2010. Using the national registry data in different time periods, we have reported the effects of epinephrine use and lactated Ringer solution use upon resuscitation outcome in patients with OHCA [14]–[16]. The results of the present study derived from the included patients are new findings.

The study was approved by the Ethics Committee at Kyushu University Graduate School of Medicine. The requirement for written informed consent was waived.

### Data collection

In Japan, 47 municipal governments provide EMS through 807 fire stations with dispatch centers [17]–[19]. All patients with OHCA who were treated by EMS personnel were transported to hospitals, excluding those with decapitation, incineration, decomposition, rigor mortis or dependent cyanosis because the Japanese guidelines do not allow EMS providers to terminate resuscitation in the field [20]. Based on the standardized Utstein style template, the Fire and Disaster Management Agency (FDMA) registered all OHCA cases in Japan in a prospective, nationwide and population-based manner. In particular, data concerning bystander cardiopulmonary resuscitation (CPR), automated external defibrillator use and the components of CPR used by EMS personnel (*i.e.*, initial rhythm, defibrillation, intubation and epinephrine use) were collected using EMS records. The EMS person responsible for each patient with OHCA met the physician who treated the patient at the hospital and collected 1-month follow-up data [21], [22]. Then, the data from the 807 fire stations with dispatch centers in the 47 prefectures were electronically integrated into the national registry system on the FDMA database server.

### Ambulance crew

An ambulance crew consisted of three emergency providers, including at least one emergency life-saving technician, but no physician. The certifying paramedic curriculum in Japan generally includes 180 h of lectures and practice in school and experience in 30 successful cases in the operating room under the instruction of an anesthesiologist [23]. Emergency life-saving technicians are permitted to insert adjunct airways and to use semi-automated external defibrillators [17]. With approval from an online emergency physician, specially trained emergency life-saving technicians have been permitted to insert intravenous lines since July 2004, and certified emergency life-saving technicians have been permitted to administer intravenous epinephrine since April 2006 [17].

A physician who happens to be with a patient when the patient collapses outside a hospital, or who happens to be in an ambulance for the training of the ambulance crew, might be engaged in pre-hospital cardiopulmonary resuscitation (CPR) until the patient's arrival at the hospital. In this study, the criterion for the presence of a physician in the resuscitation team was whether a physician rode in an ambulance from the scene of a patient's collapse to their arrival at the hospital. The presence of a physician was completely unplanned, and his/her role was not clear. If a physician accompanies the patient in an ambulance, the physician can independently perform advanced life support (ALS) (*i.e.*, perform tracheal intubation, insert an intravenous line and/or use epinephrine). It is possible that a physician might administer ALS in addition to basic life support, such as chest compression and rescue breathing, ECG analysis or team management in an ambulance. In this study, ALS was performed in 15.08% of all cases in which a physician rode with the patient in an ambulance.

### Variables

The collected data included information on OHCA cases, CPR initiated by a bystander, life support provided by EMS personnel and patient outcome. Patients who survived cardiac arrest were followed for up to 1 month after the event, and data on the survival and neurological and physical status were collected. Neurological outcomes 1 month after successful resuscitation were evaluated using the cerebral performance category (CPC) scale, with five categories (1, good cerebral performance; 2, moderate cerebral disability; 3, severe cerebral disability; 4, coma or vegetative state; 5, death), and the overall performance category (OPC) scale, also with five categories (1, no or mild neurological disability; 2, moderate neurological disability; 3, severe neurological disability; 4, coma or vegetative state; 5, death) [21], [22], [24]. At 1 month after the cardiac event, the EMS person responsible for the patient with OHCA contacted the physician in charge of that patient and collected CPC and OPC data. These data were entered into the same national database.

The variables used in the study are listed in Table 1. In particular, the etiology of cardiac arrest (*i.e.*, cardiac or non-cardiac) was determined clinically by the physician in charge, with the aid of EMS personnel. Because an automated external defibrillator (AED) analyzed the patient's rhythm automatically and delivered a shock only when it detected ventricular fibrillation (VF), the patient's first recorded rhythm was regarded as VF when laypersons delivered shocks with the use of a public access AED. Additionally, the category of VF included ventricular tachycardia (VT).

**Table 1:** Baseline characteristics of patients with OHCA according to the presence of a physician in the ambulance car: National data between 2005 and 2010 in Japan (n = 619,928).

Variable	Physician in ambulance car (n = 17,186)	No physician in ambulance car (n = 602,742)	*p*
*(OHCA patients)*			
Cases by year			
2005, No. (%)	2486 (14.47)	89169 (14.80)	<0.001
2006, No. (%)	2571 (14.96)	93796 (15.56)	
2007, No. (%)	2491 (14.49)	98044 (16.27)	
2008, No. (%)	2694 (15.68)	102960 (17.08)	
2009, No. (%)	3258 (18.96)	105147 (17.45)	
2010, No. (%)	3683 (21.43)	113535 (18.84)	
Age, yr (SD)	69.60 (17.16)	72.96 (16.31)	<0.001
Sex (male), No. (%)	10700 (62.26)	352130 (58.42)	<0.001
Bystander eyewitness (%)	10152 (59.07)	242462 (40.23)	<0.001
Relationship of bystander to patient (family member), No. (%)	3708 (21.58)	126556 (21.00)	<0.001
Origin of OHCA			
Cardiac, No. (%)	9442 (54.94)	334468 (55.49)	0.35
Non-cardiac, No. (%)	7744 (45.06)	268274 (44.51)	
*(CPR initiated by bystander)*			
Chest compression, No. (%)	7683 (44.70)	230718 (38.66)	<0.001
Rescue breathing, No. (%)	3732 (21.72)	80174 (13.49)	<0.001
Use of public-access AED, No. (%)	350 (2.04)	3318 (0.56)	<0.001
*(Life support by EMS personnel)*			
Emergency life-saving technician in ambulance car, No. (%)	16436 (95.64)	602742 (100.00)	<0.001
ALS by MD, No. (%)	12474 (72.58)	81015 (13.44)	<0.001
Time from call to arrival at scene, min (SD)	7.38 (4.26)	7.31 (3.74)	<0.05
Time from call to arrival at hospital, min (SD)	39.77 (21.00)	32.15 (13.07)	<0.001
First documented rhythm			
VF/pulseless VT, No. (%)	2174 (12.65)	44017 (7.30)	<0.001
PEA/Asystole, No. (%)	15012 (87.35)	558725 (92.70)	
Defibrillation by EMS personnel, No. (%)	3172 (18.46)	63943 (10.67)	<0.001
Use of ALS device (laryngeal mask/adjunct airway/tracheal tube), No. (%)	6764 (39.36)	274367 (45.54)	<0.001
Insertion of intravenous line, No. (%)	4878 (28.38)	135443 (22.58)	<0.001
Epinephrine use, No. (%)	2801 (16.30)	37345 (6.25)	<0.001
*(Endpoints)*			
ROSC before hospital arrival, No. (%)	4220 (24.55)	39459 (6.55)	<0.001
1-month survival after cardiac arrest, No. (%)	1917 (11.15)	30036 (4.98)	<0.001
1-month CPC (good performance/moderate disability), No. (%)	967 (5.63)	14465 (2.40)	<0.001
1-month OPC (no or mild neurological disability/moderate neurological disability), No. (%)	962 (5.60)	14324(2.38)	<0.001

Note: With respect to all variables in the table, missing values ranged from 5 to 10,998.

The endpoints consisted of four types (Table 1). These endpoints were return of spontaneous circulation (ROSC) before hospital arrival, survival at 1-month after cardiac arrest, 1-month survival with CPC category 1 or 2, and 1-month survival with OPC category 1 or 2 [21], [22], [24]. Of these, survival at 1-month after cardiac arrest was regarded as a major outcome measure.

### Statistical analysis

The data that met the criteria concerning the patient age, time course and the presence of a physician in the ambulance were analyzed (*n* = 619,928). Using data for all 619,928 patients, three types of unconditional logistic regression models were fitted using the endpoints listed in Table 1 as the dependent variables. Several variables have been shown to be predictors of the resuscitation outcome in patients with OHCA, including age, sex, bystander eyewitness, relationship of bystander to patient, bystander chest compression, bystander rescue breathing, use of public-access AED by the bystander, first documented rhythm and time from call to arrival at the scene [25]. The effect of the presence of a physician was examined using three types of analysis models that differed in the degree of controlling for the effects of the covariates. Specifically, the first model did not control for the effects of the covariates, the second model controlled for the effects of the predictor variables, and the third model controlled for all covariates. Variability existed with respect to the quality of emergency medical services; thus, 46 dummy variables were introduced to 47 prefectures in Japan, and the third model controlled for the effect of the areas. In each analysis model, three types of subjects were used. The first model type used the total number of subjects. To exclude the effect of the number of ambulance crew members (*i.e.*, three vs. four), the second model type used cases with three-member ambulance crews. To evaluate the effect of the presence of a physician in the ambulance, the third model type used cases that excluded subjects in which the physician performed CPR but did not ride in the ambulance. Of the four primary outcome variables, 1-month survival was used for the sample size calculation. With an actual 1-month survival rate of 11.15% in the group accompanied by a physician in the ambulance and 4.98% in the group without the presence of a physician (Table 1), 619,928 samples per group provided a power level of 100.00% with a type I error of 1% [26].

The presence of a physician in the ambulance was not assigned randomly in the patient population; therefore, we developed a propensity score for the presence of a physician in the ambulance and controlled for potential confounding and selection biases [27]. Without regard to patient outcome, the propensity score for the physician presence in the ambulance was determined using multivariate logistic regression analysis. Specifically, a full non-parsimonious logistic regression model was fitted with the physician presence in the ambulance as the dependent variable; the independent variables included all study variables except for endpoint variables plus dummy variables for the 47 prefectures in Japan (*i.e.*, 46 variables). A propensity score for the presence of a physician in an ambulance was calculated from the logistic regression equation for each patient. This propensity score represented the probability of the physician presence in the ambulance. Using the propensity score in the SAS Macro program by Parsons et al. [28], cases in which a physician rode in the ambulance were matched to unique control cases in which a physician was not present. Using data on the propensity-matched subjects, five types of conditional logistic regression models were fitted, with each of the endpoint variables listed in Table 2 as the dependent variable. To precisely evaluate the effect of the presence of a physician on a patient's outcome, the effect of every covariate needed to be controlled [29]. In the propensity-matched sample, there were significant differences between groups with and without a physician with respect to several variables. In addition, several variables have been reported previously to be predictors of the resuscitation outcome in patients with OHCA [25]. Thus, the effect of the presence of a physician was examined using five types of analysis model that differed in the degree of controlling for the effects of the covariates. Specifically, the first model does not control for the effects of covariates; the second model controls for the effects of propensity; the third model controls for the effects of propensity and significant variables in propensity-matched samples; the fourth model controls for the effects of propensity, significant variables in propensity-matched samples and variables reported to be predictors of the resuscitation outcomes [25]; and the fifth model controls for the effects of propensity and all covariates. Of the four primary outcome variables, 1-month survival was used for sample size calculation. With an actual 1-month survival rate of 15.61% in the group accompanied by a physician and 12.66% in the group not accompanied by a physician (Table 2), 9,231 samples for each group provided a power level of 100.00% with a type I error of 1% [26].

**Table 2:** Baseline characteristics of propensity-matched OHCA patients according to the presence of a medical doctor in the ambulance car.

Variable	Physician in ambulance car (n = 9,231)	No physician in ambulance car (n = 9,231)	*p* value
*(OHCA patients)*			
Cases by year			
2005, No. (%)	1437 (15.57)	1436 (15.56)	0.92
2006, No. (%)	1475 (15,98)	1470 (15.92)	
2007, No. (%)	1094 (11.85)	1066 (11.55)	
2008, No. (%)	1459 (15.81)	1450 (15.71)	
2009, No. (%)	1804 (19.54)	1863 (20.18)	
2010, No. (%)	1962 (21.25)	1946 (21.08)	
Age, yr (SD)	69.41 (16.93)	69.44 (17.70)	0.92
Sex (male), No. (%)	5894 (63.85)	5905 (63.97)	0.87
Bystander eyewitness, No. (%)	9217 (99.85)	9211 (99.78)	0.30
Relationship of bystander to patient (family member), No. (%)	3444 (37.31)	3286 (35.60)	0.02
Origin of OHCA			
Cardiac, No. (%)	5108 (55.34)	5137 (55.65)	0.67
Noncardiac, No. (%)	4123 (44.66)	4094 (44.35)	
*(CPR initiated by bystander)*			
Chest compression, No. (%)	4003 (43.36)	4044 (43.81)	0.54
Rescue breathing, No. (%)	2079 (22.52)	2099 (22.74)	0.73
Use of public-access AED, No. (%)	242 (2.62)	245 (2.65)	0.89
*(Life support by EMS personnel)*			
Emergency life-saving technician in ambulance car, No. (%)	8855 (95.93)	9231 (100.00)	<0.001
ALS by MD, No. (%)	6965 (75.45)	1454 (15.75)	<0.001
Time from call to arrival at scene, min (SD)	7.33 (4.25)	7.36 (4.12)	0.60
Time from call to arrival at hospital, min (SD)	41.11 (21.88)	39.54 (23.94)	<0.001
First documented rhythm			
VF/pulseless VT, No. (%)	1558 (16.88)	1523 (16.50)	0.49
PEA/Asystole, No. (%)	7673 (83.12)	7708 (83.50)	
Defibrillation by EMS personnel, No. (%)	2158 (23.38)	2136 (23.14)	0.70
Use of ALS device (laryngeal mask/adjunct airway/tracheal tube), No. (%)	3690 (39.97)	3658 (39.63)	0.63
Insertion of intravenous line, No. (%)	2554 (27.67)	2640 (28.60)	0.16
Epinephrine use, No. (%)	1665 (18.04)	1693 (18.34)	0.59
*(Endpoints)*			
ROSC before hospital arrival, No. (%)	2774 (30.05)	1661 (17.99)	<0.001
1-month survival after cardiac arrest, No. (%)	1441 (15.61)	1169 (12.66)	<0.001
1-month CPC (good performance/moderate disability), No. (%)	753 (8.16)	716 (7.76)	0.31
1-month OPC (no or mild neurological disability/moderate neurological disability), No. (%)	753 (8.16)	702 (7.60)	0.16

The significance level for all tests was *p*<0.05 (two-sided). All analyses were performed using the SAS software (ver. 8.2; SAS Institute, Cary, NC, USA).

## Results

**Figure 1:**
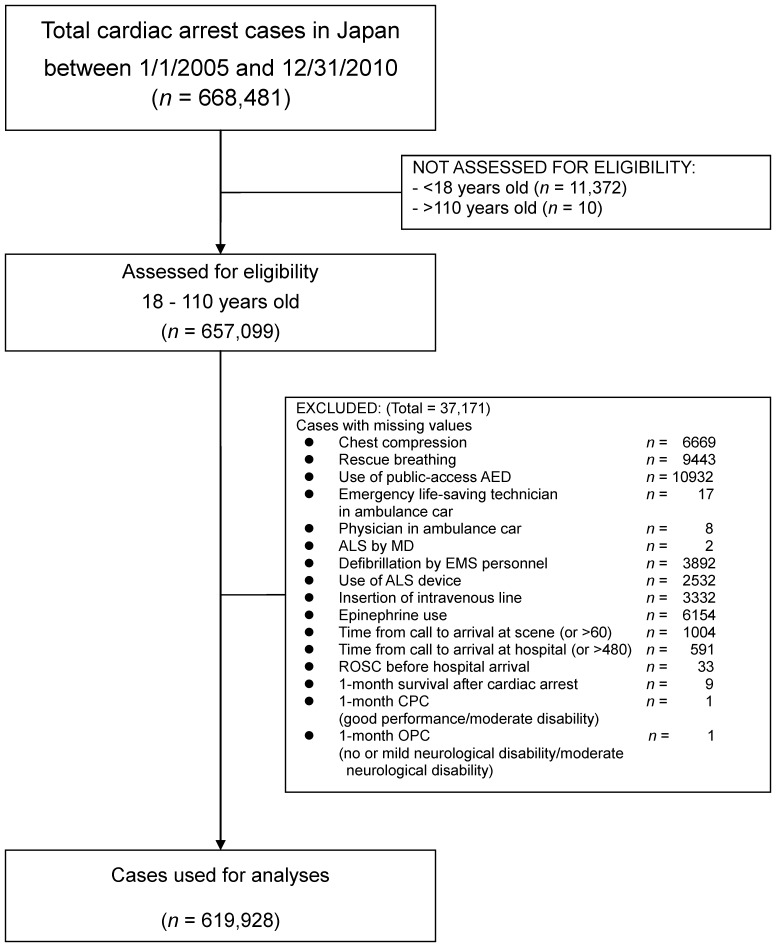
OHCA cases included in the study.

Between 1 January 2005 and 31 December 2010, 668,481 cardiac arrests occurred. Of these cases, 619,928 patients with OHCA met the inclusion criteria (Fig 1). The types of missing values in 37,171 cases are indicated in Figure 1. With respect to the ‘total cardiac arrest cases in Japan between 1/1/2005 and 12/31/2010’ (*n* = 668,481), ‘assessed for eligibility 18–110 years old’ (*n* = 657,099) and ‘cases used for analyses’ (*n* = 619,928), the mean ages of patients with OHCA were 71.75±18.42, 72.34±16.58 and 72.91±16.32 years, respectively. The numbers (percentages) of female patients with OHCA among the three groups were 277,580 (41.4%), 277, 580 (42.2%) and 273,111 (44.1%), respectively. The numbers (percentages) of eyewitness cases were 272,331 (40.7%), 272,331 (41.4%), and 268,513 (43.3%), respectively. The numbers (percentages) of cases of cardiac origin were 369,131 (55.2%), 369,131 (56.2%) and 365, 608 (59.0%), respectively.

Table 1 shows the baseline characteristics of patients with OHCA according to the presence of a physician in the ambulance. There was no significant difference in the prevalence of OHCA by origin for the two groups (*p* = 0.35). With respect to the other variables listed in Table 1, there was a significant difference between those who rode in an ambulance car with a physician and those who did not. A significant difference was observed between those who rode in an ambulance with a physician and those who did not with respect to the prevalence of variables that are known predictors of the resuscitation outcome in patients with OHCA (*i.e.*, age, sex, bystander eyewitness, relationship of bystander to patient, bystander chest compression, bystander rescue breathing, use of public-access AED by a bystander, first documented rhythm and time from call to arrival at the scene) [25]. The time from the call to arrival at the scene was 7.38 and 7.31 min, respectively, among those who rode in an ambulance with a physician and those who did not.

### Physician's ambulance car ride and patient survival

Table 3 summarizes survival outcome based on a physician's presence during an ambulance car ride among three types of subjects. Among all subjects, with respect to the four types of outcome variables, there was a significant and positive association between a physician's presence and the four outcome measures in the unadjusted, adjusted for selected variables, and adjusted for all covariates models (all *p*<0.001), except for 1-month survival in the adjusted for all covariates model, which showed a significant and negative association. Among the three ambulance crew groups, there was a significant and positive association between a physician's presence during an ambulance ride and ROSC before hospital arrival in the unadjusted, adjusted for selected variables, and adjusted for all covariates models (all *p*<0.001). Among subjects, excluding cases for which a physician did advanced life support (ALS) but did not ride in the ambulance, there was a significant and positive association between a physician's presence during the ambulance ride and the four outcome measures in the unadjusted, adjusted for selected variables, and adjusted for all covariates models (all *p*<0.01), except for 1-month survival, CPC (1 or 2) and OPC (1 or 2) in the adjusted for all covariates model.

**Table 3:** Unconditional logistic regression analyses of a physician in the ambulance car and outcome among patients with OHCA.

	ROSC		1-mo survival		CPC (1 or 2)		OPC (1 or 2)	
	OR (95% CI)	*p*	OR (95% CI)	*p*	OR (95% CI)	*p*	OR (95% CI)	*p*
Unadjusted								
Total (n = 619,928)	4.65 (4.48–4.82)	<0.001	2.39 (2.28–2.51)	<0.001	2.43 (2.27–2.59)	<0.001	2.44 (2.28–2.61)	<0.001
3-member ambulance crew (n = 603,493)^a^	3.54 (2.96–4.24)	<0.001	1.58 (1.20–2.06)	<0.01	1.70 (1.18–2.44)	<0.01	1.71 (1.19–2.47)	<0.01
Excluded cases, in which physician did ALS^b^ but did not ride in ambulance car (n = 538,913)	4.67 (4.50–4.84)	<0.001	2.40 (2.28–2.52)	<0.001	2.42 (2.26–2.58)	<0.001	2.43 (2.27–2.60)	<0.001
Adjusted for selected variables^c^								
Total (n = 609,865)	3.60 (3.46–3.74)	<0.001	1.59 (1.51–1.68)	<0.001	1.37 (1.27–1.48)	<0.001	1.38 (1.28–1.48)	<0.001
3-member ambulance crew (n = 593,512)^a^	3.54 (2.96–4.24)	<0.001	1.07 (0.80–1.43)	0.63	1.02 (0.69–1.51)	0.93	1.03 (0.70–1.53)	0.87
Excluded cases, in which physician did ALS^b^ but did not ride in ambulance car (n = 530,633)	3.61 (3.47–3.75)	<0.001	1.58 (1.50–1.66)	<0.001	1.35 (1.25–1.45)	<0.001	1.36 (1.26–1.46)	<0.001
Adjusted for all covariates^d^								
Total (n = 569,793)	2.66 (2.54–2.79)	<0.001	0.91 (0.85–0.97)	<0.01	0.80 (0.73–0.88)	<0.001	0.82 (0.74–0.90)	<0.001
3-member ambulance crew (n = 554,019)^a^	2.02 (1.63–2.50)	<0.001	0.65 (0.47–0.91)	<0.05	0.51 (0.33–0.79)	<0.01	0.52 (0.34–0.81)	<0.01
Excluded cases, in which physician did ALS^b^ but did not ride in ambulance car (n = 498,223)	1.90 (1.75–2.07)	<0.001	1.11 (0.98–1.25)	0.11	1.11 (0.95–1.31)	0.20	1.11 (0.94–1.31)	0.20

OR: odds ratio; CI: confidence interval.

a: Cases for which both an emergency life-saving technician (ELST) and a physician (MD) were in the ambulance car were excluded.

b: Advanced life support.

c: Selected variables included age, sex, bystander eyewitness, relationship of bystander to patient, bystander chest compression, bystander rescue breathing, use of public-access AED by bystander, first documented rhythm, and time from call to arrival at the scene for a model with ROSC as a dependent variable. As for other models, ROSC and the above selected variables were adjusted.

d: All covariates included all variables except for endpoint variables listed in Table 1 plus 46 dummy variables for the 47 prefectures in Japan for a model with ROSC as a dependent variable. As for other models, ROSC, all variables except for endpoint variables listed in Table 1, and 46 dummy variables for the 47 prefectures in Japan were adjusted.

### Physician's ambulance car ride and survival in propensity-matched patients

To calculate the propensity score, a full non-parsimonious logistic regression model was fitted. This model yielded a *c* statistic of 0.85, which indicated a good ability to differentiate between cases in which a physician rode in the ambulance car and cases without a physician present. The propensity scores ranged from 0.003 to 1.000, which indicated that the probability of a physician's presence was between 0.003 and 1. In the study, 9,231 patients who were with a physician in the ambulance were matched to 9,231 patients who were without a physician in the ambulance (Table 2). With respect to every predictor variable, except for the relationship of bystander to patient, the presence of an emergency life-saving technician in the ambulance car, ALS by a medical doctor (MD), and time from call to scene arrival, no significant differences were detected between groups with and without a physician.

Table 4 summarizes survival outcomes based on a physician's presence in the ambulance among propensity-matched patients. Of the four types of endpoint variables, a positive association was detected between a physician's presence during the ambulance ride and ROSC in all models (all *p*<0.001). In terms of an association between a physician's presence during the ambulance ride and 1-month survival, a positive association was detected in all models, with the exception of the unadjusted models (*p*<0.05 for the last model and *p*<0.001 for the remaining models). In terms of an association between a physician's presence during the ambulance ride and CPC (1 or 2) or OPC (1 or 2), a positive association was detected in the models that adjusted for propensity and significant variables in the propensity-matched samples in Table 2, which adjusted for propensity and significant variables in the propensity-matched samples in Table 2 and selected variables, and which adjusted for propensity and all covariates (*p*<0.05 for the last model and *p*<0.001 for the remaining models) (Table 4).

**Table 4:** Conditional logistic regression analyses of a medical doctor in the ambulance car and outcome among propensity-matched patients with OHCA (n = 18,462).

	ROSC		1-month survival		CPC (1 or 2)		OPC (1 or 2)	
	OR (95% CI)	*p*	OR (95% CI)	*p*	OR (95% CI)	*p*	OR (95%CI)	*p*
Method								
Unadjusted	1.18 (1.12–1.23)	<0.001	1.05 (0.99–1.10)	0.08	1.01 (0.96–1.06)	0.80	1.01 (0.96–1.06)	0.73
Adjusted for propensity	1.97 (1.83–2.11)	<0.001	1.28 (1.18–1.39)	<0.001	1.06 (0.95–1.18)	0.31	1.08 (0.97–1.20)	0.16
Adjusted for propensity and significant variables in propensity-matched samples listed in Table 2^a^	1.83 (1.64–2.04)	<0.001	1.52 (1.34–1.72)	<0.001	1.48 (1.25–1.74)	<0.001	1.48 (1.26–1.75)	<0.001
Adjusted for propensity, significant variables in propensity-matched samples listed in Table 2, and selected variables^b^	1.76 (1.57–1.98)	<0.001	1.49 (1.29–1.71)	<0.001	1.44 (1.18–1.76)	<0.001	1.43 (1.17–1.75)	<0.001
Adjusted for propensity and all covariates^c^	1.84 (1.63–2.07)	<0.001	1.29 (1.04–1.61)	<0.05	1.54 (1.03–2.29)	<0.05	1.50 (1.01–2.24)	<0.05

OR: odds ratio; CI: confidence interval.

a: Significant variables in propensity-matched samples in listed Table 2 included relationship of bystander to patient, emergency life-saving technician in ambulance car, ALS by MD, and time from call to arrival at hospital.

b: Selected variables included age, sex, bystander eyewitness, relationship of bystander to patient, bystander chest compression, bystander rescue breathing, use of public-access AED by bystander, first documented rhythm, and time from call to arrival at the scene for a model with ROSC as a dependent variable. As for other models, ROSC and the above selected variables were adjusted.

c: All covariates included all variables except for endpoint variables listed in Table 1 plus 46 dummy variables for the 47 prefectures in Japan for a model with ROSC as a dependent variable. As for other models, ROSC, all variables except for endpoint variables listed in Table 1, and 46 dummy variables for the 47 prefectures in Japan were adjusted.

## Discussion

Based on a valid propensity analysis that controls for the effects of selection bias and confounding factors, we are the first to reveal that a physician's presence during the ambulance car ride is independently associated with short- and long-term outcome. Since previous findings were inconsistent, we believe that our findings are important both theoretically and practically. As for the possible positive effect of a physician's presence during an ambulance car ride upon resuscitation outcome, several possible reasons have been suggested. First, the presence of a physician improves outcomes of cardiac arrest because advanced procedures, such as airway management and epinephrine use, can be done by the physician [6]. However, this possibility contradicts previous studies that found both intubation and epinephrine use to be independent predictors of poor outcome in patients with OHCA [15], [30], [31]. Second, physicians are more likely to comply with treatment guidelines and possess up-to-date knowledge than other ambulance personnel [6], [32]. According to our analysis of CPR by physician's presence status, the following was revealed (see the Supporting Information, Table S1): (1) of procedures for which effectiveness was demonstrated (*i.e.*, chest compression and defibrillation), multiple procedures were more frequently performed in the physician group than in the non-physician group; (2) a set of procedures that should be performed simultaneously (*i.e.*, initial shockable rhythm and defibrillation) were more frequently performed in the physician group than in the non-physician group; and (3) no use of ineffective procedures (*i.e.*, epinephrine use [15] and ALS devices [31]), along with the use of effective procedures, was more frequently used in the physician group than in the non-physician group. Thus, the present findings might be due to the second reason. In addition, physicians are reportedly more efficient in managing procedures such as ECG analysis and team management [6]. The presence of a defined team leader, with experience and knowledge to provide oversight during resuscitation, could explain the increased focus on quality of care. Although this possibility might be applicable to the present study, we could not verify this point in the study. In summary, the better outcome in the group of patients accompanied by a physician in the ambulance might be related to the quality of medical care due to the physician's up-to-date knowledge and better compliance with treatment guidelines.

Since analysis based on all subjects was influenced by the effects of selection bias and confounding factors, such results were not consistent with those based on propensity-matched subjects, except for the association between the presence of a physician and ROSC (Tables 2 and 4). However, since the results based on different analysis models agreed, the association between the presence of a physician and ROSC found among all subjects can be trusted. It is possible that the improved quality of CPR might not be due to the specific presence of a physician, as it might be due to having four persons on the PMA versus three persons on the first responding non-PMA [6]. Among subjects whose crew member number was three, there was a significant and positive association between a physician's presence and ROSC before hospital arrival (Table 3). These results suggest that the improved quality of CPR was, at least in part, due to the presence of a physician specifically. Of the 602,742 cases without a physician during the ambulance ride, there were 81,015 cases for which a physician did ALS. Among the remaining subjects, excluding cases for which a physician did ALS but did not ride in the ambulance car, there was a significant and positive association between a physician's presence during the ambulance ride and ROSC before hospital arrival (Table 2). Thus, a physician's presence might have a positive effect on resuscitation outcome in patients with ROSC.

Regarding the analytical method used in this study, because the proportions of ROSC before hospital arrival differed between groups with and without a physician in an ambulance (Tables 1 and 3), it might be speculative to suggest an additional effect on the other three types of endpoints [*i.e.*, 1-month survival, CPC (1, 2) and OPC (1, 2)]. However, long-term survival cannot be achieved without first restoring circulation. In addition, ROSC is used widely as a measure of short-term survival. Thus, in this study, ROSC was entered into the analysis model as an independent variable when evaluating the association between the presence of the physician in an ambulance car and long-term survival.

Several limitations and caveats to our study must be acknowledged. First, we performed propensity analysis and made a rigorous adjustment for selection bias and confounding factors, which would be expected with a standard multivariate analysis [33]. Nevertheless, since a physician's presence during an ambulance car ride was not assigned by random allocation, we need to acknowledge that we can only partially control and adjust for factors actually measured. Second, data on in-hospital CPR after hospital arrival were not included in the analysis. It is possible that our findings might have been influenced by differences in in-hospital resuscitation, such as hypothermia [34] and mechanical chest compression devices [35], between those who were with a physician during the ambulance car ride and those who were not. Although the quality of in-hospital resuscitation might influence 1-month survival, we could not control for the effects of such factors. Third, the specialty(ies) of the physicians who rode in ambulance cars was unknown, and it is probable that a physician who was adept at CPR of OHCA patients rode in the car. If this was the case, then the association between the physician's presence and resuscitation outcome might have been over-estimated. However, we could not control for the effects of such factors. Fourth, to evaluate the effect of the presence of a physician in an ambulance on the resuscitation outcome of patients with OHCA, data are required regarding the survival rate after hospital discharge and 6 months later. However, we could not evaluate the effect of physician presence in an ambulance on these outcome variables due to the lack of relevant data.

In summary, despite the limitations of this study, the associations between a physician's presence during an ambulance car ride and increased short- and long-term outcomes were consistent. Additional analysis also indicated that the presence of a physician was beneficial for CPR of patients with OHCA. Our findings should be verified by studies that include in-hospital resuscitation data.

## Supporting Information

Table S1 **Components of CPR by type of emergency crew (n = 619,928).**
(DOCX)

## References

[1] Lossius HM, Soreide E, Hotvedt R, Hapnes SA, Eielsen OV, et al (2002) Prehospital advanced life support provided by specially trained physicians: is there a benefit in terms of life years gained? Acta Anaesthesiologica Scandinavica 46: 771–778. 12139529 10.1034/j.1399-6576.2002.460703.x

[2] Skogvoll E, Bjelland E, Thorarinsson B (2000) Helicopter emergency medical service in out-of-hospital cardiac arrest - a 10-year population-based study. Acta Anaesthesiologica Scandinavica 44: 972–979. 10981575 10.1034/j.1399-6576.2000.440813.x

[3] Edelson DP, Litzinger B, Arola V, Kim S, Lauderdale DS, et al (2008) Improving in-hospital cardiac arrest process and outcome with performance debriefing. Arch Intern Med 168: 1063–1069. 18504334 10.1001/archinte.168.10.1063

[4] Hampton JR, Dowling M, Nicholas C (1997) Comparison of results from a cardiac ambulance manned by medical or non-medical personnel. Lancet 1: 526–529. 10.1016/s0140-6736(77)91384-8 65621

[5] Baxt WG, Moody P (1987) The impact of a physician as part of the aeromedical prehospital team in patients with blunt trauma. JAMA 257: 3246–3250. 3586248

[6] Olasveegen TM, Lund-Kordahl I, Steen PA, Sunde K (2009) Out-of hospital advanced life support with or without a physician: effects on quality of CPR and outcome. Resuscitation 80: 1248–1252. 19709795 10.1016/j.resuscitation.2009.07.018

[7] Botker MT, Bakke SA, Christensen EE (2009) A systematic review of controlled studies: do physicians increase survival with prehospital treatment? Scandinavian Journal of Trauma, Resuscitation and Emergency Medicine 17: 12 (doi:01.1186/1757-7241-17-12) 10.1186/1757-7241-17-12 PMC2657098 19265550

[8] Yen ZS, Chen YT, Ko PCI, Ma MHM, Chen SC, et al (2006) Cost-effectiveness of different advanced life support providers for victims of out-of-hospital cardiac arrest. Journal of Formosan Medial Association 105: 1001–1007. 10.1016/S0929-6646(09)60284-9 17185242

[9] Dickinson ET, Schneider RM, Verdile VP (1997) The impact of prehospital physicians on out-of-hospital nonasystolic cardiac arrest. Prehosp Emerg Care 1: 132–135. 9709354 10.1080/10903129708958805

[10] Soo LH, Gray D, Young T, Huff N, Skene A, et al (1999) Resuscitation from out-of-hospital cardiac arrest: is survival dependent on who is available at the scene? Heart 81: 47–52. 10220544 10.1136/hrt.81.1.47 PMC1728906

[11] Sipria A, Talvik R, Korgvee A, Sarapuu S, Oopik A (2000) Out-of-hospital resuscitation in Tartu: effect of reorganization of Estonian EMS system. Am J Emerg Med 8: 469–473. 10.1053/ajem.2000.7350 10919542

[12] Frandsen F, Nielsen JR, Gram L, Larsen CF, Jorgensen HR, et al (1991) Evaluation of intensified prehospital treatment in out-of-hospital cardiac arrest: survival and cerebral prognosis. The Odense ambulance study. Cardiology 79: 256–264. 1782642 10.1159/000174888

[13] Mitchell RG, Brady W, Guly UM, Pirrallo RG, Robertson CE (1997) Comparison of two emergency response systems and their effect on survival from out of hospital cardiac arrest. Resuscitation 35: 225–229. 10203400 10.1016/s0300-9572(97)00072-5

[14] Abe T, Nagata T, Hasegawa M, Hagihara A (2012) Life support related to survival after out-of-hospital cardiac arrest in infants: a population study in Japan. Resuscitation 83: 612–618. 22289682 10.1016/j.resuscitation.2012.01.024

[15] Hagihara A, Abe T, Hasegawa M, Nagata T, Wakata Y (2012) Intravenous epinephrine use before hospital arrival and survival among out-of-hospital cardiac arrest patients. JAMA 307(11): 1161–1168. 22436956 10.1001/jama.2012.294

[16] Hagihara A, Hasegawa M, Abe T, Wakata Y, Nagata T, et al (2013) Prehospital Lactated Ringer's Solution Treatment and Survival in Out-of-Hospital Cardiac Arrest: A Prospective Cohort Analysis. PLoS Med 10(2): e1001394. 23431275 10.1371/journal.pmed.1001394 PMC3576391

[17] Kitamura T, Iwami T, Kawamura T, Nagao K, Tanaka H, et al (2010) Nationwide public-access defibrillation in Japan. N Engl J Med 362: 994–1004. 20237345 10.1056/NEJMoa0906644

[18] Ogawa T, Akahane M, Koike S, Tanabe S, Mizoguchi T, et al (2011) Outcomes of chest compression only CPR versus conventional CPR conducted by lay people in patients with out of hospital cardiopulmonary arrest witnessed by bystanders: nationwide population based observational study. BMJ 342: c7106 doi: 10.1136/bmj.c7106 21273279 10.1136/bmj.c7106

[19] Kitamura T, Iwami T, Kawamura T, Nagao K, Tanaka H, et al (2010) Conventional and chest-compression-only cardiopulmonary resuscitation by bystanders for children who have out-of-hospital cardiac arrests: a prospective, nationwide, population-based cohort study. Lancet 375: 1347–54. 20202679 10.1016/S0140-6736(10)60064-5

[20] Japanese guidelines for emergency care and cardiopulmonary resuscitation. 3rd ed. (2007) Tokyo: Health Shuppansha.

[21] Cummins RO, Chamberlain DA, Abramson NS, Allen M, Baskett P, et al (1991) Recommended guidelines for uniform reporting of data from out-of-hospital cardiac arrest: the Utstine style- a statement for health professionals from a task force of the American Heart Association, the European Resuscitation Council, the Heart and Stroke Foundation of Canada, and the Australian Resuscitation Council. Circulation 84: 960–75. 1860248 10.1161/01.cir.84.2.960

[22] Jacobs I, Nadkami V, Bahr J, Finn J, Halperin H, et al (2004) Cardiac arrest and cardiopulmonary resuscitation outcome reports: update and simplification of the Utstein template for resuscitation registries: a statement for healthcare professionals from a task force of the International Liaison Committee on Resuscitation. Circulation 110: 3385–97. 15557386 10.1161/01.CIR.0000147236.85306.15

[23] Takei Y, Enami M, Yachida T, Ohta K, Inaba H (2010) Tracheal intubation by paramedics under limited indication criteria may improve the short-term outcome of out-of-hospital cardiac arrests with noncardiac origin. Journal of Anesthesia 24: 716–725. 20577765 10.1007/s00540-010-0974-6

[24] Cummins RO, Chamberlain DA, Hazinski MF, Nadkarni V, Kloeck W, et al (1997) Recommended guidelines for reviewing, reporting, and conducting research on in-hospital resuscitation; The n-hospital “Utstein style”. A statement for healthcare professionals from the American Heart Association, the European Resuscitation Council, the Heart and Stroke Foundation of Canada, the Australian Resuscitation Council, and the Resuscitation Councils of South Africa. Circulation 95: 2213–39. 9133537 10.1161/01.cir.95.8.2213

[25] Sasson C, Rogers MAM, Dahl J, Kellermann AL (2010) Predictors of survival from out-of-hospital cardiac arrest. A systematic ewview and meta-analysis. Circ Cardiovasc Qual Outcomes 3: 63–81. 20123673 10.1161/CIRCOUTCOMES.109.889576

[26] Hsieh FT, Block DA, Larsen MD (1998) A simple method of sample size calculation for linear and logistic regression. Stat Med 17: 1623–1634. 9699234 10.1002/(sici)1097-0258(19980730)17:14<1623::aid-sim871>3.0.co;2-s

[27] Joffe MM, Rosenbaum PR (1999) Invited commentary: propensity scores. Am J Epidemiol 150: 327–33. 10453808 10.1093/oxfordjournals.aje.a010011

[28] Parsons LS, Ovation Research Group, Seattle, WA. Paper 214-26: Reducing bias in a propensity score matched-pair sample using greedy matching techniques. Available: http://www2.sas.com/proceedings/sugi26/p214-26.pdf#search=sas%2Cpropensitymatching%2Cparsons%2CSUGI26 Accessed 2013 November 26.

[29] Gum PA, Thamilarasan M, Watanabe J, Blackstone EH, Lauer MS (2001) Aspirin use and all-cause mortality among patients being evaluated for known or suspected coronary artery disease. A propensity analysis. JAMA 286(10): 1187–1194. 11559263 10.1001/jama.286.10.1187

[30] Holmberg M, Holmberg S, Herlitz J (2002) Low chance of survival among patients requiring adrenaline epinephrine or intubation after out-of-hospital cardiac arrest in Sweden. Resuscitation 54: 37–45. 12104107 10.1016/s0300-9572(02)00048-5

[31] Hasegawa K, Hiraide A, Chang Y, Brown DFM (2013) Association of prehospital advanced airway management with neurologic outcome and survival in patients with out-of-hospital cardiac arrest. JAMA 309: 257–266. 23321764 10.1001/jama.2012.187612

[32] Kirves H, Skrifvars MB, Vahakuopus M, Ekstrom K, Martikainen M, et al (2007) Adherence to resuscitation guidelines during prehospital care of cardiac arrest patients. Eur J Emerg Med 14: 75–81. 17496680 10.1097/MEJ.0b013e328013f88c

[33] Joffe MM, Rosenbaum PR (1999) Invited commentary: propensity scores. Am J Epidemiol 150: 327–33. 10453808 10.1093/oxfordjournals.aje.a010011

[34] Bernard SA, Gray TW, Buist MD, Bernard SA, Gray TW, et al (2002) Treatment of comatose survivors of out-of-hospital cardiac arrest with induced hypothermia. N Engl J Med 346: 549–56. 11856794 10.1056/NEJMoa003289

[35] Grogaard HK, Wik L, Eriksen M, Brekke M, Sunde K (2007) Continuous mechanical chest compressions during cardiac arrest to facilitate restoration of coronary circulation with percutaneous coronary intervention. J Am Coll Cardiol 50: 1093–94. 17825721 10.1016/j.jacc.2007.05.028

