# Supplementary material for: Physician Presence in an Ambulance Car Is Associated with Increased Survival in Out-of-Hospital Cardiac Arrest: A Prospective Cohort Analysis
Source: PLoS One. 2014 Jan 8;9(1):e84424. doi: 10.1371/journal.pone.0084424 (PMC3885569; doi:10.1371/journal.pone.0084424)
Supplement: Table S1 — Components of CPR by type of emergency crew (n = 619,928). (DOCX) [file pone.0084424.s001.docx]

| **Supporting Information:Table S1. Components of CPR by type of emergency crew (n = 619,928).** | | | |
| --- | --- | --- | --- |
|  | MD in crews (N = 17,186) | No MD in crews (N = 602,742) | *p*^a^ |
| 1. Chest compression + defibrillation by EMS personnel | 1,585 (9.22%) | 27,444 (4.55%) | <0.0001 |
| 2. Initial shockable rhythm + defibrillation by EMS personnel | 2,033 (11.83%) | 43,307 (7.18%) | <0.0001 |
| 3. Initial shockable rhythm + defibrillation by EMS personnel + chest compression | 1,133 (6.59%) | 18,825 (3.12%) | <0.0001 |
| 4. No epinephrine use + no use of ALS devices^b^ | 9,509 (55.33%) | 348,957 (57.89%) | 0.08 |
| 5. No epinephrine use + no use of ALS devices^b^ + chest compression | 4,263 (24.81%) | 122,595 (20.34%) | <0.0001 |
| 6. No epinephrine use + no use of ALS devices^b^ + defibrillation by EMS personnel | 1,607 (9.35%) | 32,863 (5.45%) | <0.0001 |
| a: Chi-square test.  b: Laryngeal mask/adjunct airway/tracheal tube. |  |  |  |
